# CRISPR-epigenetic crosstalk: From bidirectional regulation to therapeutic potential

**DOI:** 10.1016/j.csbj.2025.10.031

**Published:** 2025-10-19

**Authors:** Yixiao Wei, Jia Sun, Ruigong Zhu

**Affiliations:** a School of Pharmacy, Nanjing University of Chinese Medicine, Nanjing City 210023, China; b National Resource Center for Mutant Mice, GemPharmatech Co., Ltd, Nanjing City 210023, China; c State Key Laboratory of Functions and Applications of Medicinal Plants, Guizhou Medical University, Guiyang City 550014, China

**Keywords:** CRISPR-Cas, Epigenetic modifications, Gene editing technology, Precision medicine, Bidirectional regulation

## Abstract

Recent advances in epigenetics have elucidated the pivotal roles of epigenetic modifications in genomic regulation and disease pathogenesis. Concurrently, CRISPR-based technologies have transcended conventional gene-editing applications and have emerged as powerful tools for target gene screening, chromatin imaging, and epigenetic modulation. Notably, epigenetic landscapes substantially influence the CRISPR editing efficiency, whereas CRISPR itself can reshape epigenetic states, forming a dynamic CRISPR-Epigenetics Regulatory Circuit. This review systematically examines the bidirectional interplay between CRISPR systems and epigenetic modifications, emphasizing their collective impact on genome-editing precision, disease progression, and therapeutic development. Existing studies have predominantly focused on the application of CRISPR in epigenetic modifications or the impact of epigenetic landscapes on CRISPR, exhibiting unidirectional characteristics. However, accumulating evidence suggests a bidirectional interaction between the two. Here, a transformative “CRISPR-Epigenetics Regulatory Circuit” model is synthesized and presented, supported by three pivotal breakthroughs: demonstrating CRISPR as an active epigenetic programmer, synthesizing the epigenetic preconditioning therapeutic paradigm, and elucidating the first predictive mathematical model (EPIGuide). Further exploration of this circuit is expected to enhance CRISPR performance, optimize *sgRNA* design via epigenetic predictive models, and pioneer sequential epigenetic or gene editing therapies.

## 1 Introduction

Epigenetic modifications, including DNA methylation, histone post-translational modifications, and noncoding RNA-mediated regulation, represent crucial layers of genomic control that dynamically influence gene expression without altering the underlying DNA sequence. These mechanisms fundamentally shape cellular identity, differentiation, and tissue homeostasis, and their dysregulation is intimately linked to disease pathogenesis. For instance, in cancer, hypermethylation of tumor suppressor gene promoters can lead to their transcriptional silencing, facilitating uncontrolled proliferation and metastasis [1].

In parallel, the Clustered Regularly Interspaced Short Palindromic Repeats (CRISPR) system has evolved from an adaptive bacterial immune system into a versatile biotechnological platform. While initially renowned for its gene-editing capabilities, CRISPR technology has rapidly expanded into functional genomics, chromatin imaging, and notably, epigenetic engineering. A key innovation in this regard is the nuclease-deactivated Cas9 (dCas9), which serves as a programmable DNA-binding module. By fusing dCas9 with various epigenetic effector domains, researchers can now perform precise locus-specific modifications. For example, Zhang et al. [2] developed a dCas9-based editor that recruits a citrullination enzyme to specific histone arginine residues, enabling targeted histone citrullination and subsequent gene regulation.

Notably, the relationship between CRISPR and epigenetics is not unidirectional. The cellular epigenetic landscape exerts a profound influence on CRISPR activity. DNA methylation, for instance, can impair Cas9 binding and reduce editing efficiency, particularly when target sites reside within highly methylated CpG islands [3]. Similarly, histone modifications modulate chromatin accessibility: repressive marks such as H3K27me3 compact chromatin and hinder Cas9 access, whereas acetylated histones often correlate with enhanced editing outcomes. These observations have been quantitatively supported by machine learning approaches. Algorithms such as EPIGuide demonstrate that integrating epigenetic features, including chromatin accessibility and histone modification states, can improve sgRNA efficacy prediction by 32–48 % over sequence-based models alone [4].

This reciprocity, where CRISPR systems can rewrite epigenetic states, and pre-existing epigenetic states constrain or facilitate CRISPR activity, forms the basis of what we term the “CRISPR-Epigenetics Regulatory Circuit”. This dynamic, closed-loop model challenges the conventional view of CRISPR as a passive tool and reframes it as both an effector and a target of epigenetic regulation. Understanding this circuit offers new avenues to improve editing precision, predict disease-relevant epigenetic alterations, and develop novel therapeutic strategies that leverage bidirectional feedback.

This review will focus on the interplay between CRISPR systems and epigenetics in mammalian systems and its therapeutic implications. We begin by outlining the functional architecture of major CRISPR systems, then examine CRISPR-driven epigenome engineering and the reciprocal impact of epigenetics on CRISPR efficiency. Finally, we synthesize evidence for the regulatory circuit model and discuss its translational potential, including sequential editing strategies and computational modeling, while also addressing persistent challenges such as epigenetic memory durability and tissue-specific variability. Readers interested in CRISPR-epigenetic applications in plants or other systems are directed to specialized reviews [5].

## 2 CRISPR-Cas system

### 2.1 Functional architecture and evolutionary diversity of CRISPR-Cas systems

The CRISPR/Cas system, an adaptive immune mechanism in prokaryotes, uses CRISPR arrays, composed of conserved repeats interspersed with variable spacers derived from invasive nucleic acids, to generate CRISPR-derived RNAs(*crRNA*s) for sequence-specific pathogen neutralization[6], [7], [8]. Upon reinfection, *crRNA* complexes with Cas nucleases recognize complementary viral sequences via protospacer-adjacent motifs (PAM)-dependent targeting, triggering the cleavage of foreign *DNA* or *RNA* [9], [10]. Although CRISPR loci exhibit structural plasticity, their functional core lies in spacer acquisition and *crRNA*-guided interference. Evolutionary diversification has produced two major classes: class 1 systems (types I, III, and IV) utilize multi-protein effector complexes, whereas class 2 systems (types II, V, and VI) rely on single effector proteins, such as Cas9 and Cas12a [11], [12]. Class 2 systems dominate genome-editing applications owing to their simplicity. Additionally, almost all of the characterized systems belong to Class 1; however, fewer than one in ten of these are functionally programmable [13].

Notably, the CRISPR toolkit exhibits considerable diversity and is subject to continuous discovery. The systems compared in Table 1 (Cas9, Cas12a, Cas13) represent the most widely adopted and well-characterized effectors for genome and transcriptome engineering. However, ongoing discovery efforts are revealing a wealth of additional systems, such as the hypercompact Cas12f [14]and the RNA-targeting Type III-E Cas7–11 [15], with unique properties that may be repurposed for novel epigenetic applications in the future.

**Table 1:** The specific mechanism of the CRISPR system.

**Feature**	**CRISPR-Cas9**	**CRISPR-Cas12a**	**CRISPR-Cas13**
**Target Molecule**	DNA	DNA	RNA
**PAM Sequence**	5'-NGG−3′	5'-TTTN−3′	No PAM
**crRNA Structure**	tracrRNA and crRNA complex	crRNA	crRNA
**Cutting Site**	3–4 bp upstream of PAM	18–23 bp downstream of PAM	Any position of RNA target
**Off-target Effects**	Higher	Lower	Higher (RNA targeting specificity is lower)

### 2.2 Mechanism of class 2 effectors

Cas9 and Cas12a exemplify the functional specialization of Class 2 systems. Cas9 (type II) requires a dual-*RNA* complex for *DNA* recognition and generates blunt double-stranded (DSBs) in PAMs (NGG). In contrast, Cas12a (type V) operates via a single *crRNA*, cleaves *DNA* with staggered ends, and recognizes T-rich PAMs (TTTV), thus enabling distinct genomic targeting landscapes. Recent engineering breakthroughs, such as hypercompact Cas MINI, have overcome the size limitations of conventional Cas9 by leveraging structural insights to delete non-essential domains while maintaining activity and enhancing viral vector compatibility for therapeutic delivery [16]. However, persistent challenges include PAM restrictions, off-target effects in heterochromatic regions, and inefficient base editing in low-division cells. System-level optimization, such as combining PAM-expanding variants, improves spatial precision and tissue-specific editing fidelity [17].

### 2.3 CRISPR-Cas mechanisms and epigenetic crosstalk

The CRISPR-Cas system, originating as an adaptive immune mechanism in prokaryotes, operates through three conserved stages: spacer acquisition, crRNA biogenesis, and target interference [18]. During adaptation, Cas1-Cas2 complexes integrate protospacer sequences from invading nucleic acids into the host CRISPR array, guided PAMs to avoid self-targeting [19]. These spacers are transcribed and processed into mature crRNAs, which guide Cas nucleases to cleave complementary foreign genetic elements during reinfection. Among Class 2 systems, Cas9 and Cas12a represent two widely used effectors with distinct mechanisms. Cas9 depends on a sgRNA and recognizes NGG PAMs to generate blunt-ended DSBs via its HNH and RuvC domains [20], [21], [22], [23]. In contrast, Cas12a processes its own crRNA array, recognizes T-rich PAMs, and induces staggered DSBs with additional collateral single-stranded DNA cleavage activity [24], [25], [26], [27].

When deployed in eukaryotic cells, CRISPR activity is strongly influenced by the native chromatin environment. Chromatin accessibility, DNA methylation, and histone modifications significantly modulate Cas protein binding and editing efficiency. For example, heterochromatin marked by high DNA methylation or repressive histone modifications such as H3K9me3 impedes Cas9 access, while open chromatin regions enriched with H3K27ac facilitate efficient editing. This interplay underscores a bidirectional relationship: not only does CRISPR manipulate genetic sequences, but the epigenetic landscape also shapes its operational efficacy.

To exploit CRISPR for epigenetic engineering without inducing DNA breaks, catalytically inactive variants have been developed. The dead Cas9 (dCas9), generated by point mutations that inactivate the HNH and RuvC nuclease domains, serves as a programmable DNA-binding scaffold [28]. By fusing dCas9 with epigenetic effector domains, such as methyltransferases, acetyltransferases, or demethylases, targeted epigenome editing can be achieved. This approach, broadly termed Epi-CRISPR, enables precise manipulation of DNA methylation, histone modifications, and chromatin architecture at specific loci [29], [30]. Similarly, nuclease-deficient Cas12a (dCas12a) has been repurposed for transcriptional regulation and chromatin remodeling in AT-rich genomic regions [31]. For instance, dCas9-p300 fusions can activate silenced tumor suppressor genes via histone acetylation [32], while dCas12a-TET1 constructs promote enhancer demethylation to direct cell differentiation [33]. A major challenge, however, lies in the stability of edited epigenetic states, which are often reversed by endogenous enzymatic activities or diluted through DNA replication, underscoring the need for sustained epigenetic memory systems [34].

The epigenetic context also influences DNA repair pathway choice following CRISPR-induced DSBs. Error-prone non-homologous end joining (NHEJ) is favored in heterochromatic regions, whereas homologous-directed repair (HDR) is more efficient in transcriptionally active euchromatin [35], [36]. This bias poses a challenge for therapeutic genome editing, as many disease-relevant loci reside within repressive chromatin domains. To overcome this limitation, combinatorial strategies are being explored, including the use of chromatin-modulating drugs or engineered HDR enhancers alongside CRISPR machinery [37]. Epigenetic context dependency is notably reduced in more recent CRISPR-derived tools such as base and prime editors, which operate without generating DSBs.

Notably, the relationship between chromatin state and editing efficiency can be harnessed therapeutically. A compelling example is epigenetic preconditioning, wherein targeted chromatin remodeling is performed prior to genetic editing. Qian et al. [38] demonstrated this approach by first using dCas9-TET1 to demethylate the *PD-L1* enhancer, converting it from a repressive to an accessible chromatin state. This preconditioning significantly enhanced the efficiency of subsequent Cas9-mediated *PD-L1* knockout, leading to robust antitumor immunity and tumor regression in mouse models. This paradigm illustrates how deliberate epigenetic modulation can be integrated with CRISPR technology to overcome chromatin barriers and optimize therapeutic outcomes.

## 3 CRISPR-driven epigenome engineering

CRISPR-based epigenome engineering has revolutionized our ability to interrogate and therapeutically modulate gene regulatory networks. By leveraging nuclease-deficient Cas proteins fused with epigenetic effectors, researchers can now implement precise transcriptional control and chromatin remodeling without altering DNA sequence.

CRISPR interference (CRISPRi) and CRISPR activation (CRISPRa) systems have become indispensable for mapping functional epigenetic elements. These platforms have uncovered critical 3D chromatin hubs and master regulators of disease pathogenesis. For example, CRISPRi screens identified MYB as a key regulator of leukemia progression through its role in maintaining chromatin looping [39], while CRISPRa-mediated activation of specific enhancers has enabled direct reprogramming of fibroblasts into cardiovascular progenitor cells [40]. Large-scale CRISPR screens targeting chromatin regulators have further revealed novel mediators of tissue regeneration and cell fate determination. Key findings include the identification of *Baz2a* and *Baz2b* as regulators of liver regeneration [41] and the discovery that targeting *Oct4* or *Sox2* enhancers via CRISPRa facilitates pluripotent stem cell reprogramming through chromatin state resetting.

The therapeutic potential of epigenetic editing is being explored across diverse disease contexts. In hematological disorders, CRISPR-Cas9 knockout of the *BCL11A* enhancer in hematopoietic stem cells has demonstrated clinical efficacy in elevating fetal hemoglobin levels for treating β-hemoglobinopathies [42], [43]. In oncology, multiple approaches have shown promise: dCas9-KRAB fusions inhibit prostate cancer growth through targeted transcriptional repression [44]; *USP48* knockout synergizes with hypomethylating agents in acute myeloid leukemia [45]; and dCas9-p300-mediated histone acetylation suppresses metastasis in colorectal cancer by modulating cellular plasticity [46].

Beyond DNA-targeting systems, CRISPR-based RNA epigenetic editing platforms enable precise manipulation of RNA modifications such as m6A, m1A, and m5C [47], [48]. These tools have revealed critical roles for epitranscriptomic regulation in cancer progression, including METTL3-mediated m6A modification of PD-L1 mRNA that facilitates immune evasion [49]. Similarly, CRISPR systems have been deployed to target noncoding RNAs, silencing oncogenic lncRNAs via dCas9-repressor fusions [50] or activating tumor-suppressive lncRNAs through CRISPRa approaches [51].

The clinical translation of CRISPR-based therapies is advancing rapidly, primarily through genetic editing approaches that establish important safety and efficacy benchmarks. Notable examples include PD-1 knockout T cells for solid tumors [52] and *BCL11A* enhancer editing for sickle cell disease [43]. While these interventions target DNA sequence, they provide crucial groundwork for future epigenetic editing trials. The growing understanding of the CRISPR-epigenetics regulatory circuit, coupled with improvements in editor specificity and delivery systems, suggests that programmable epigenetic therapies may represent the next frontier in precision medicine.

**Fig. 1:**
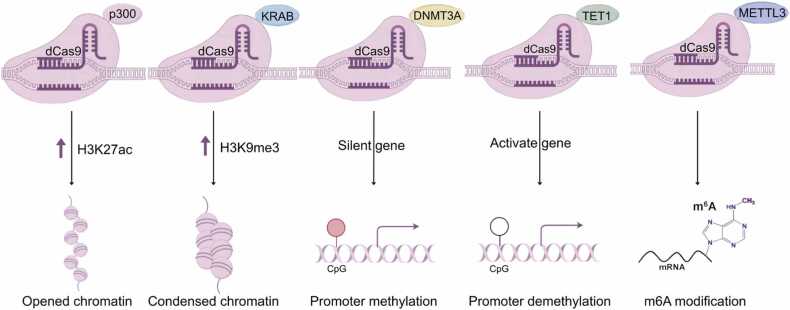
The application of CRISPR in epigenetic modifications.

In Fig. 1, we condense the current applications of CRISPR in epigenetics. And Table 2 lists the applications of major CRISPR systems in clinical trials. In Table 3, we summarize the types of CRISPR enzymes used in different tumor diseases.

**Table 2:** The applications of major CRISPR systems in clinical trials.

**Type**	**Disease model**	**References**
CRISPR-Cas9	Transfusion-dependent β-thalassemia (TDT) and sickle cell disease (SCD)	[53]
	Sickle cell disease	[54], [55]
	Edit T cells of cancer patients	[56]
	Transthyretin amyloidosis	[57]
	Anti-leukemia	[58]
	Refractory herpetic stromal keratitis (HSK)	[59]
	Hereditary angioedema	[60], [61]
	Transfusion-dependent β-thalassemia (TDT)	[62], [63]
	Transthyretin Amyloid Cardiomyopathy (ATTR-CM)	[64]
	Non-small cell lung cancer	[65]
	B-cell acute lymphoblastic leukemia	[66]
	Necrotizing myopathy, diffuse cutaneous systemic sclerosis	[67]
	Metastatic colorectal cancer	[68]
	T-cell lymphoma	[69]
	Retinal degeneration	[70]
	Aggressive B-cell non-Hodgkin lymphoma	[71]
	Editing T cells to treat tumors	[72], [73]
	Acute myeloid leukemia （AML）	[74]
	Ovarian cancer	[75]
	Pancreatic ductal adenocarcinoma	[76]
CRISPR-Cas12	COVID−19 test, nucleic acid test	[77], [78], [79], [80], [81], [82], [83], [84], [85], [86], [87], [88], [89], [90], [91], [92], [93]
	OMEGA gene editing, AVV delivery system	[94]
	DETECTR detects human papillomavirus	[95]
	Biosensor	[96]
	Programmed DNA Targeting	[97]
	Detect monkeypox virus	[98], [99]
	Targeted RNA	[100], [101]
	HBV detection	[102], [103]
	Rotavirus detection	[104**]**
	Heparin detection	[105**]**
	Detection of methicillin-resistant Staphylococcus aureus (MRSA)	[106**]**
	Detection of Mycobacterium tuberculosis complex	[107**]**
	Detect Wolbachia	[108**]**
	Detecting drug-resistant genes of Plasmodium falciparum	[109**]**
	Norovirus detection	[110**]**
	Klebsiella pneumoniae	[111**]**
	HIV test	[112**]**
	Respiratory pathogen detection	[113**]**
	Detect hepatitis C virus	[114**]**
	Detect DNA	[115**]**
	Pneumonia caused by Pneumocystis jirovecii	[116**]**
CRISPR-Cas13d	Embryonic knockdown	[117**]**
	T cell knockdown	[118**]**
	RNA knockdown	[119**]**
	Hypertrophic cardiomyopathy	[120**]**
	Anti-SARS-CoV−2 novel coronavirus	[121], [122], [123]
	Anti-Seneca Valley Virus（SVV）	[124**]**
	Detect proteins	[125**]**
	Cancer treatment	[126**]**
	Screening breast cancer-related lncRNAs	[127**]**
	Targeting Huntington's disease (HD) RNA	[128**]**
	Bladder cancer	[129], [130]
	Inhibit HIV	[131**]**
	Chronic apical periodontitis	[132**]**
	Selective elimination of uveal melanoma	[133**]**
	Targeting metastatic prostate cancer	[134**]**
	Kidney injury	[135**]**
	Anti-tumor	[136**]**
	Influenza virus	[137**]**
	Triple-negative breast cancer immunotherapy	[138**]**
	Treating gerbil hepatitis E	[139**]**
CRISPR-Cas3	Urinary tract infection induced by Escherichia coli	[140**]**

**Table 3:** The types of CRISPR enzymes used in different tumor diseases.

**Application fields**	**CRISPR types**	**Typical targeted tumors**
Genome-wide screening	CRISPR-Cas9 (knockout, CRISPRi, CRISPRa)	Various solid tumors and hematological tumors
Targeting oncogenes	CRISPR-Cas9 (knockout)	HPV-related cervical cancer/head and neck cancer, hematological tumors
	Base Editors (CRISPR-dCas9)	Tumors carrying specific point mutations
	Prime Editor (CRISPR-Cas9)	Tumors carrying various point mutations and small fragment insertions/deletions
Repair tumor suppressor genes	CRISPR-Cas9	Tumors with mutations in tumor suppressor genes such as p53, PTEN, and BRCA1/2
CAR-T/TCR-T cell modification	CRISPR-Cas9 (knockout)	Hematological tumors and solid tumors
Liquid biopsy and early screening	CRISPR-Cas12a/Cas13a	Various tumors (used for detecting mutations in circulating tumor DNA, HPV viral DNA)

## 4 Reciprocal effects of epigenetics on the CRISPR system

The functional outcome of CRISPR-mediated genome editing is profoundly influenced by the native epigenetic landscape of the target cell. This section systematically examines how key chromatin features, including nucleosome positioning, DNA methylation, and histone modifications, govern CRISPR activity and DNA repair pathway choice, and discusses emerging strategies to overcome these epigenetic constraints.

The physical compaction of chromatin represents a fundamental barrier to CRISPR activity. Densely packed nucleosomes within heterochromatic regions sterically hinder the binding of Cas9-sgRNA complexes to their target DNA sequences, significantly reducing cleavage efficiency [141], [142]. In contrast, euchromatic regions characterized by sparse nucleosome occupancy and high chromatin accessibility permit robust Cas9 binding and editing. While nucleosomes can completely block Cas9 activity in purified biochemical systems, editing remains partially feasible in living cells due to transient "breathing" of nucleosomal DNA and active remodeling by cellular chromatin remodeling complexes [143**]**. Genome-wide studies consistently demonstrate a strong positive correlation between chromatin accessibility and CRISPR editing efficiency [144**]**.

Beyond general accessibility, specific epigenetic modifications fine-tune CRISPR outcomes through distinct mechanisms: DNA Methylation. While not directly preventing Cas9-DNA binding, CpG methylation in promoter or enhancer regions recruits methyl-binding proteins and associated repressor complexes, which compact local chromatin structure and indirectly reduce editing efficiency [145**]**. Off-target Cas9 binding sites are also strongly enriched in open chromatin regions with specific DNA methylation patterns. Histone Modifications. Histone acetylation promotes an open chromatin state and significantly enhances both Cas9 binding efficiency and HDR [146**]**. This effect can be therapeutically amplified using histone deacetylase (HDAC) inhibitors, which increase global acetylation levels and have been shown to elevate Cas9 binding by over 50 % [147**]**. Conversely, repressive histone methylation marks maintain chromatin compaction and favor error-prone NHEJ over HDR [148], [149]. Notably, some anti-CRISPR proteins exploit this regulatory layer by acetylating Cas proteins themselves, impairing their DNA-binding capacity, a modification reversible by host deacetylases [150**]**.

The deterministic role of epigenetics has driven the development of sophisticated computational tools that integrate multi-omics features to predict sgRNA efficacy. Early tools like CHOPCHOP relied primarily on sequence context, whereas contemporary machine learning frameworks, including DeepCRISPR [151**]**, CRISPRscan [152**]**, and EPIGuide, leverage epigenetic features such as chromatin accessibility, histone modification maps, and DNA methylation patterns. These epigenome-aware models improve sgRNA efficacy prediction accuracy by 32–48 % compared to sequence-only approaches, and they are particularly valuable for selecting targets in repressive chromatin environments. A systematic comparison of these tools confirms that epigenetic integration is indispensable for accurate outcome prediction [153**]**.

The understanding of epigenetic constraints has inspired therapeutic strategies to transiently manipulate the chromatin landscape for improved editing outcomes. Pharmacological interventions, such as HDAC inhibitors to enhance HDR [154**]** or EZH2 inhibitors to reduce repressive H3K27me3 marks [155**]**, can create a more permissive environment for CRISPR editing. Genetic approaches have also been explored; for instance, knockdown of the lncRNA LINC01664 disrupts the SIRT1-NBS1-RAD51 complex, shifting DNA repair balance from HDR toward NHEJ and thereby sensitizing tumor cells to CRISPR-induced genotoxic stress [156], [157], [158]. These strategies exemplify the translation of mechanistic insights into practical methods for optimizing CRISPR efficacy in therapeutically relevant contexts. In Table 4, the key epigenetic features, their impacts on CRISPR activity, and potential molecular mechanisms are summarized.

**Table 4:** The key epigenetic features, their impacts on CRISPR activity, and potential molecular mechanisms.

**Epigenetic characteristics**	**Impact on CRISPR activity**	**Potential molecular mechanisms**
H3K4me3	Promote	Recruitment of chromatin remodeling factors; Chromatin accessibility
H3K9me3	Inhibit	Chromatin condensation
H3K27me3	Inhibit	Chromatin condensation
H3K36me3	Slightly promoting	Chromatin is relatively open
Histone acetylation	Promote	Neutralize the charge; Recruit “writer” proteins
CpG island methylation	Inhibit	Steric hindrance
Open chromatin	Strongly promote	Direct exposure of DNA
Closed chromatin	Strongly inhibit	Masked DNA sequence

## 5 Synthesizing the CRISPR-epigenetics regulatory circuit

The extensive evidence presented in the preceding sections reveals that the interaction between CRISPR systems and the epigenome extends beyond unidirectional causality, forming instead a dynamic, self-reinforcing CRISPR-Epigenetics Regulatory Circuit. This model posits that CRISPR activity is both shaped by and actively reshapes the epigenetic landscape, creating feedback loops that critically influence editing outcomes and therapeutic efficacy.

The circuit model is supported by multiple lines of experimental evidence demonstrating functional reciprocity. First, pre-existing epigenetic states exert direct control over CRISPR efficiency: heterochromatin impedes Cas9 access, while euchromatin enhances it. Second, CRISPR systems themselves function as potent epigenetic programmers, dCas9-effector fusions enable precise manipulation of DNA methylation, histone modifications, and higher-order chromatin structure. The most compelling validation comes from sequential editing strategies, exemplified by the PD-L1 enhancer study, where dCas9-TET1-mediated demethylation created an open chromatin state that dramatically enhanced the efficiency of subsequent Cas9-mediated genetic editing at the same locus. This demonstrates a direct causal link where an initial CRISPR to Epigenetics event directly facilitates a subsequent Epigenetics to CRISPR event, encapsulating the core circuit principle.

This circuit model provides a rational framework for overcoming a major challenge in genome editing: the inherent resistance of heterochromatic regions. It transforms the epigenetic barrier from a passive obstacle into an active therapeutic target. The strategy of epigenetic preconditioning, using dCas9-based editors to remodel chromatin into a more permissive state prior to genetic intervention, has been successfully applied not only in oncology but also in enhancing gene correction in repressive loci. Furthermore, pharmacological potentiation of the circuit, such as using HDAC inhibitors to increase chromatin accessibility and boost HDR efficiency, provides a readily translatable approach to modulate circuit dynamics for improved therapeutic outcomes.

## 6 Conclusion and outlook

**Fig. 2:**
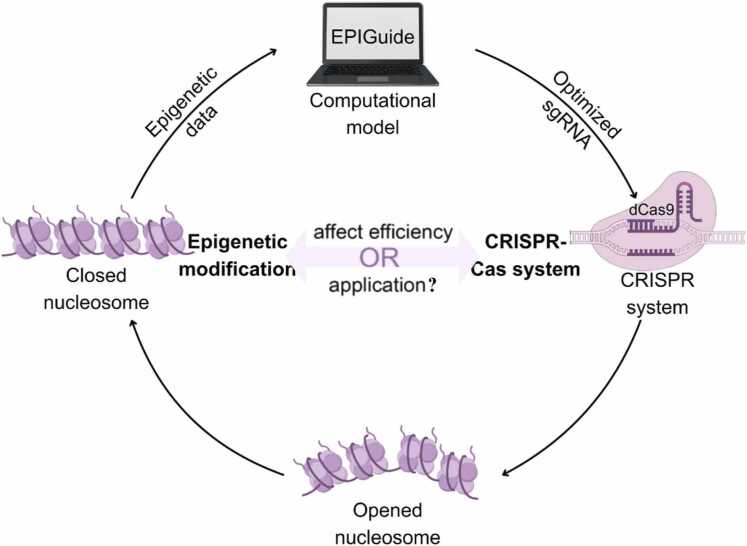
Speculations on the Interaction between CRISPR and Epigenetic Modifications.

This review synthesizes evidence supporting the CRISPR-Epigenetics Regulatory Circuit model (Fig. 2), which conceptualizes CRISPR systems as both effectors and targets of epigenetic regulation. Key findings establishing this bidirectional relationship include: pharmacological HDAC inhibition enhancing Cas9 binding efficiency through chromatin decompaction [154**]**, CRISPR-induced HDAC6 dysfunction altering broader epigenetic control [158**]**, and the synergistic enhancement of homology-directed repair through epigenetic modulator combinations [159**]**. The integration of computational tools such as EPIGuide, which improves sgRNA efficacy prediction by incorporating epigenetic features, further validates the functional significance of this regulatory circuit [160**]**.

The therapeutic potential of this paradigm is exemplified by epigenetic preconditioning strategies, where initial chromatin remodeling, as demonstrated in the PD-L1 enhancer study, facilitates subsequent efficient genetic editing. Furthermore, combining CRISPR systems with pharmacological epigenetic modulators represents a promising translational approach. Preclinical studies, including USP48 knockout synergizing with hypomethylating agents in leukemia models [161**]**, provide a rationale for future clinical trials exploring such combinations, though optimal sequencing and toxicity profiles require careful evaluation.

Several critical challenges must be addressed to advance these technologies. The durability of CRISPR-installed epigenetic marks remains uncertain, as edited states may be diluted through cell division or reversed by endogenous enzymatic activities. Efficient delivery of editing components in vivo, particularly for combinatorial approaches, presents another major hurdle. Safety considerations, including off-target effects and immune responses, are amplified in complex editing regimens. Furthermore, the context-dependent nature of the regulatory circuit necessitates tissue- and cell-type-specific optimization, as strategies effective in hematopoietic systems may not translate to solid tumors or other microenvironments.

Future investigations should prioritize longitudinal tracking of epigenetic memory persistence, single-cell analyses of circuit dynamics, and the development of next-generation algorithms capable of simulating circuit behavior using multi-omics data. Such tools would enable predictive modeling of editing outcomes, rational sgRNA design adapted to specific chromatin contexts, and the identification of therapeutic windows for epigenetic preconditioning. Addressing these challenges will accelerate the translation of CRISPR-epigenetic technologies into precision medicine applications, while simultaneously refining our fundamental understanding of gene regulation in health and disease.

## Ethics Information

Not relevant due to the nature of this manuscript.

## Declaration of Interest Statement

The authors declare that they have no known competing financial interests or personal relationships that could have appeared to influence the work reported in this paper.

## Author Contributions

Yixiao Wei, Jia Sun and Ruigong Zhu conceived the idea for this review. Yixiao Wei, Jia Sun and Ruigong Zhu wrote the manuscript and developed the summative table. Yixiao Wei, Jia Sun and Ruigong Zhu worked on formatting the manuscript for publication and made the final revisions. All the authors have read and agreed to the published version of the manuscript.

## Funding

This work was supported by grants from the 10.13039/501100001809National Natural Science Foundation of China (Grant number 82300523) and a supporting project of the National Natural Science Foundation of Nanjing University of Chinese Medicine (XPT82300523) and was financially supported by the State Key Laboratory of Functions and Applications of Medicinal Plants, 10.13039/501100010265Guizhou Medical University (Grant number JBGS-FAMP202301).

## CRediT authorship contribution statement

**Ruigong Zhu:** Visualization, Validation, Supervision, Funding acquisition. **Yixiao Wei:** Writing – review & editing, Writing – original draft, Conceptualization. **Jia Sun:** Visualization, Validation, Funding acquisition.
